# End stage renal disease patients have a skewed T cell receptor Vβ repertoire

**DOI:** 10.1186/s12979-015-0055-7

**Published:** 2015-12-14

**Authors:** Ling Huang, Anton W. Langerak, Ingrid L. M. Wolvers-Tettero, Ruud W. J. Meijers, Carla C. Baan, Nicolle H. R. Litjens, Michiel G. H. Betjes

**Affiliations:** Department of Internal Medicine, Section Nephrology and Transplantation, Erasmus University Medical Center, Room NA-523, P.O. Box 2040, 3000 CA Rotterdam, The Netherlands; Department of Immunology, Erasmus University Medical Center, Rotterdam, The Netherlands

**Keywords:** End stage renal disease, T cell receptor Vβ repertoire diversity, Age, Cytomegalovirus–IgG serostatus, CD28 null T cells

## Abstract

**Background:**

End stage renal disease (ESRD) is associated with defective T-cell mediated immunity. A diverse T-cell receptor (TCR) Vβ repertoire is central to effective T-cell mediated immune responses to foreign antigens. In this study, the effect of ESRD on TCR Vβ repertoire was assessed.

**Results:**

A higher proportion of ESRD patients (68.9 %) had a skewed TCR Vβ repertoire compared to age and cytomegalovirus (CMV) – IgG serostatus matched healthy individuals (31.4 %, *P* < 0.001). Age, CMV serostatus and ESRD were independently associated with an increase in shifting of the TCR Vβ repertoire. More differentiated CD8^+^ T cells were observed in young ESRD patients with a shifted TCR Vβ repertoire. CD31-expressing naive T cells and relative telomere length of T cells were not significantly related to TCR Vβ skewing.

**Conclusions:**

ESRD significantly skewed the TCR Vβ repertoire particularly in the elderly population, which may contribute to the uremia-associated defect in T-cell mediated immunity.

**Electronic supplementary material:**

The online version of this article (doi:10.1186/s12979-015-0055-7) contains supplementary material, which is available to authorized users.

## Background

Patients suffering from end stage renal disease (ESRD) have an impaired T-cell mediated immune system, characterized by an increased susceptibility for infections [1], a decreased response to vaccination [2–4] and a heightened risk for virus-associated cancers [5]. Loss of renal function is associated with a severe depletion of naive T cells and a shift to more differentiated memory T cells [6]. An advanced decline in thymic output and attrition of telomeres was noted in both CD4^+^ as well as CD8^+^ T cells of ESRD patients [7, 8]. These uremia-induced effects on T cells closely resemble premature T-cell aging and revealed a discrepancy of 15–20 years between the patient’s immunological age of their T cells and their chronological age [7].

A diverse (polyclonal) T cell receptor (TCR) repertoire capable of recognizing a broad range of foreign antigens is key to an effective T-cell-mediated immune response [9]. Naive T cells migrating from the thymus into the circulation, carrying CD31 antigen on their cell surface [10], possess the broadest TCR repertoire (i.e. polyclonal TCR repertoire) [11]. The memory T cells that develop upon encountering of an antigen have a repertoire that is being skewed towards particular specificities [12] but this does not automatically imply a loss of TCR diversity. Similarly, although the composition of the circulating T cells may be profoundly altered in ESRD patients, it is not known whether this invariably leads to an oligoclonal TCR repertoire reflecting a limited capacity to respond to novel encountered antigens.

Most TCRs consist of an α and a β chain and each chain is composed of a variable (V) region and a constant (C) region. In the thymus, the V region of the TCR α and β chain is generated by random gene rearrangement of variable (V) and joining (J) genes or V, diversity (D) and J genes, respectively. Contraction of TCR Vβ repertoire has been reported to occur during aging, starting from roughly 600 × 10^3^ clonal types detected per 10^6^ T cells in childhood and declining by 5 × 10^3^ clones per year [13]. The TCR Vβ repertoire diversity might also be affected by chronic antigenic stimulation [14–16]. For example, cytomegalovirus (CMV) latency may also induce contraction of the TCR Vβ repertoire as it results in a vast expansion of CMV specific T cell-exceeding 4 % of CD8^+^ T cells in immunocompetent donors [17] and these anti-CMV T cells clones were stably maintained for 5 years [18]. CMV-IgG seropositive ESRD patients have shorter telomeres within CD8^+^ T cells and an increased T cell differentiation status with higher percentages of CD57^+^ and CD28^−^ CD4^+^ and CD8^+^ memory T cells [19]. Both CMV latency and ESRD, alone or in combination, may profoundly alter the composition of the peripheral T cell compartment [19, 20].

We hypothesized that ESRD may decrease the TCR Vβ repertoire diversity and that CMV latency could further add to this loss of diversity. In this study, we therefore assessed the TCR Vβ repertoire diversity of ESRD patients relative to age- and CMV-IgG serostatus- matched healthy individuals (HI). The TCR Vβ repertoire diversity was evaluated by a qualitative multiplex DNA-based PCR of TCR Vβ-Jβ gene rearrangements, originally designed to diagnose lymphoproliferations [21]. The generated Genescan profile was used to distinguish between a Gaussian distributed (diverse or polyclonal) and a more or less skewed (narrowed or even monoclonally expanded) TCR Vβ repertoire.

## Results

### Study population characteristics

The demographic and clinical characteristics of the study population are given in Table 1. Approximately 45 % of ESRD patients received renal replacement therapy with the median dialysis time of 13 months. Twenty-one patients were within the young group (age 19 – 45 years) and 24 patients belonged to the elderly group (age 65 – 77 years).

**Table 1:** Clinical and biological characteristics of the study population

	Healthy individuals	ESRD Patients	*P* value
Number of individuals	51	45	
Age (young/eldely) (years; mean ± SD)	33.1 ± 7.6/68.0 ± 3.0	32.9 ± 9.2 / 68.3 ± 3.5	NS/NS^a^
Sex (% male)	41.2	68.9	*P* < 0.05
CMV IgG serostatus (% pos)	52.9	51.1	NS
T cells number in peripheral blood (10^6^/μl; mean ± SD)	1274 ± 304	1164 ± 260	NS
Renal replacement therapy (number; %)		44.4	
Duration of Renal replacement therapy (months; median/rang)		13/3-68	
Hemodialysis (%)		80.0	
Peritoneal dialysis (%)		15.0	
Peritoneal dialysis followed hemodialysis (%)		5.0	
Underlying kidney disease			
Nephrosclerosis/atherosclerosis/hypertensive nephropathy (%)		17.8	
Primary glomerulopathy (%)		6.7	
Diabetic nephropathy (%)		20.0	
Reflux nephropathy (%)		17.8	
Polycystic kidney disease (%)		20.0	
Other (%)		15.6	
Unknown (%)		2.2	

*Abbreviations*: *ESRD* end stage renal disease, *CMV* cytomegalovirus, *NS* not significant

^a^Age categories of young (≤45 years) and elderly ( ≥65 years) were used

### ESRD is associated with a skewed TCR Vβ repertoire

**Fig. 1:**
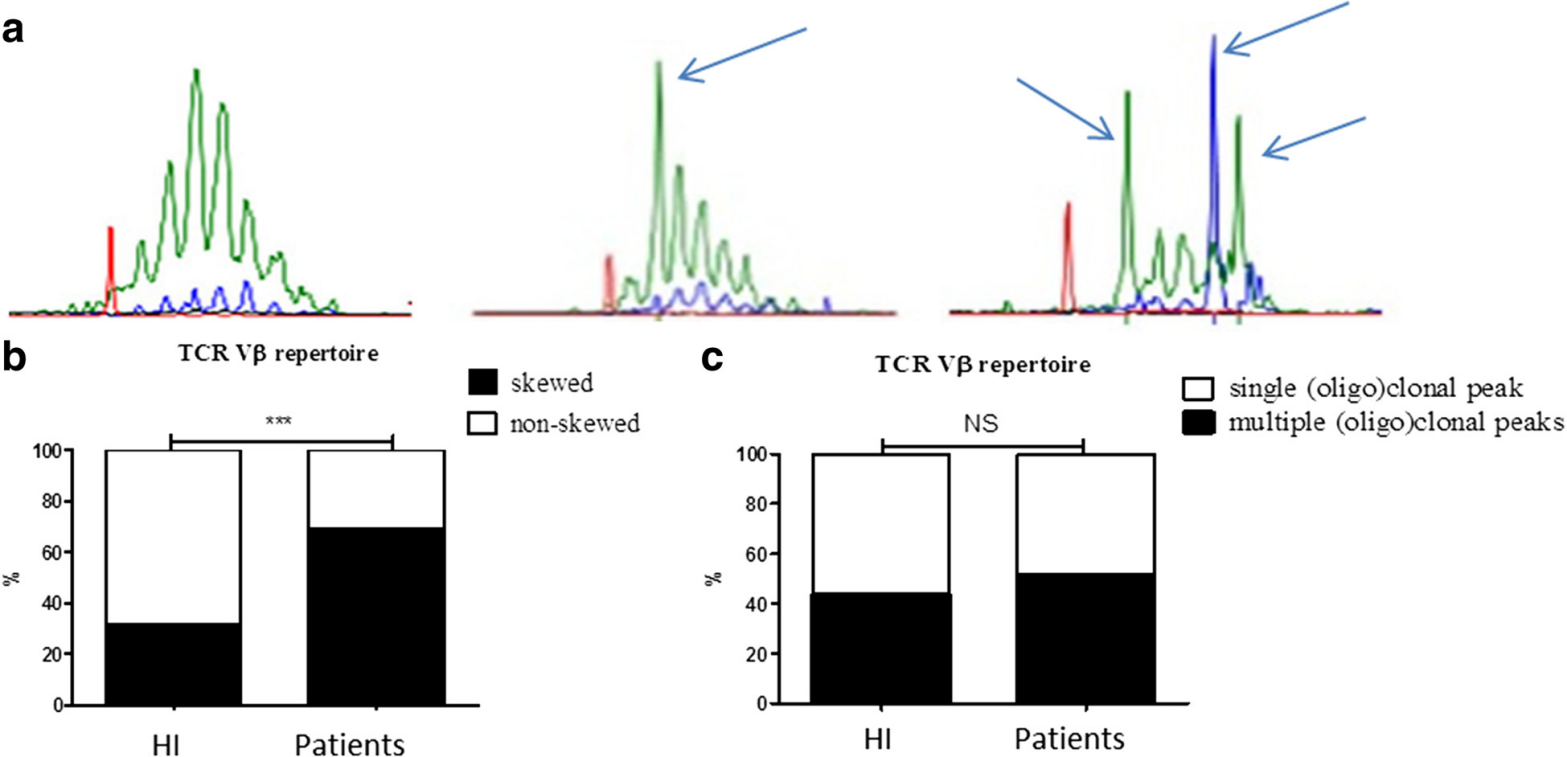
Skewed and non-skewed TCR Vβ repertoire distribution in healthy individuals (HI) and end stage renal disease (ESRD) patients. The different genescan profiles obtained following spectratyping of the DNA-based multiplexed TCR Vβ PCR samples are depicted in **a**. This reaction consists of 23 Vβ primers and 9 Jβ primers. The green profile represents the 6 Jβ primers (Jβ1.1, Jβ1.2, Jβ1.3, Jβ1.4, Jβ1.5, Jβ1.6), whereas the blue line represents the 3 Jβ primers (Jβ2.2, Jβ2.6, and Jβ2.7) in this multiplex PCR. The red profile represents the internal size standard. The left profile shows a non-skewed (polyclonal) pattern, the middle one shows a skewed pattern with a single (oligo)clonal peak (size of the oligoclonal product is 253 nucleotides (nt)) whereas the right panel depicts a skewed pattern with multiple (oligo)clonal peaks (sizes of the oligoclonal products are 252, 261 and 264 nt, respectively). The arrows indicate oligoclonal peaks within the genescan profile. **b** Frequency of ESRD patients (*n* = 45) and age-matched HI (*n* = 51) with a skewed (*closed squares*) and non-skewed (*open squares*) TCR Vβ repertoire. **c** Frequency of single (*open squares*) and multiple (oligo)clonal peaks (*closed squares*) within a skewed TCR Vβ repertoire of ESRD patients (*n* = 31) and HI (*n* = 16). P value: * <0.05; ** < 0.01; *** < 0.001; NS: not significant

A TCR Vβ repertoire was defined as non-skewed one when the spectratype of TCR Vβ repertoire on the Genescan profile showed a Gaussian distribution (Fig. 1a, left profile) or a skewed one showing an oligoclonal pattern with one (Fig. 1a middle profile) or more clonal (Fig. 1a, right profile) peak(s) on the Genescan profile. A skewed (or oligoclonal) TCR Vβ repertoire was present in a larger (*P* < 0.001) proportion (68.9 %) of the ESRD patients compared to HI (31.4 %) (Fig. 1b). Further dividing the results into single or multiple (oligo)clonal peaks did not lead to significantly different percentages in skewed TCR Vβ repertoire between the ESRD patients and HI (Fig. 1c). Percentages of individuals with multiple (oligo)clonal peaks in the skewed TCR Vβ repertoire amounted to 51.6 and 43.7 % in ESRD patients and HI, respectively (*P* > 0.05). In conclusion, ESRD is associated with skewing of the TCR Vβ repertoire.

### Both Aging and CMV serostatus affect the TCR Vβ repertoire diversity in ESRD patients

**Fig. 2:**
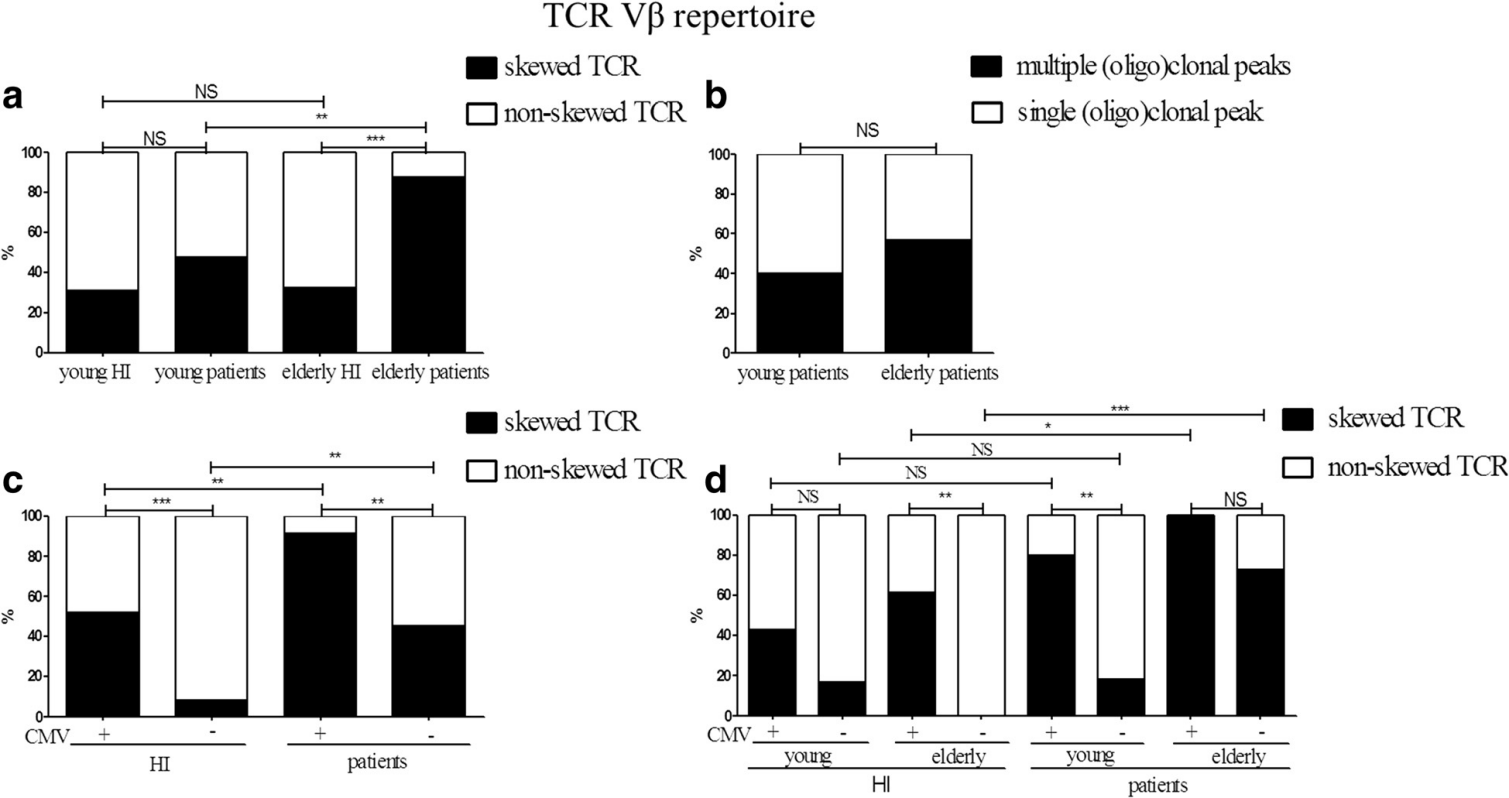
Skewed and non-skewed TCR Vβ repertoire distribution in healthy individuals (HI) and end stage renal disease (ESRD) patients dissecting the population into subgroups according to age and CMV-IgG serostatus. **a** Frequency of a skewed TCR Vβ repertoire in young (*n* = 21) and elderly (*n* = 24) ESRD patients compared to age- matched HI (young, *n* = 26 and elderly, *n* = 25). **b** Frequency of multiple (oligo)clonal peaks within a skewed TCR Vβ repertoire of young (*n* = 10) ESRD patients compared to elderly ESRD patients (*n* = 21). **c** Frequency of a skewed TCR Vβ repertoire in CMV-IgG seropositive (*n* = 23) and seronegative (*n* = 22) ESRD patients, compared to CMV-IgG seropositive (*n* = 27) and seronegative (*n* = 24) healthy individuals. **d** Frequency of a skewed TCR Vβ repertoire in young ESRD patients with CMV- IgG seropositive status (*n* = 10) or CMV-IgG seronegative status (*n* = 11) and elderly ESRD patients with CMV-IgG seropositive status (*n* = 13) or CMV-IgG seronegative status (*n* = 11), compared to age-matched CMV-IgG seropositive (young *n* = 14, elderly *n* = 13) and CMV IgG seronegative (young *n* = 12, elderly *n* = 12) HI. The closed squares represent skewed or multiple oligo(clonal) peaks within skewed TCR Vβ repertoires whereas the open squares represent non-skewed or single (oligo)clonal peaks within skewed TCR Vβ repertoires, respectively. P value: * <0.05; ** < 0.01; *** < 0.001; NS: not significant

Dissecting the ESRD and healthy study population into a young and elderly group, revealed significantly more (*P* < 0.001) skewing within the elderly ESRD patients compared to the age-matched HI, proportions were 87.5 % versus 32.0 %, respectively (Fig. 2a). In addition, an age-related skewing in TCR Vβ repertoire was observed for ESRD patients (*P* < 0.01), but not for healthy individuals (Fig. 2a). Moreover, like for the overall study population, no differences were observed between the composition of multiple and single oligoclonal peaks in young and elderly ESRD patients with a skewed TCR Vβ repertoire (Fig. 2b).

We then looked at the effect of CMV IgG seropositivity on TCR Vβ repertoire skewing. A skewed TCR Vβ repertoire was observed in 41.7 and 16.7 % of CMV-IgG seropositive and CMV-IgG seronegative HI, respectively. Approximately, 91.3 % of CMV-IgG seropositive ESRD patients had a skewed TCR Vβ repertoire whereas skewing of the TCR Vβ repertoire occurred in 45.5 % of CMV-IgG seronegative ESRD patients. CMV latency skewed (*P* < 0.01) the TCR Vβ repertoire in both HI as well as ESRD patients (Fig. 2c). Data was further analyzed taking the effect of aging into account. In young CMV-IgG seropositive and negative patients, percentage of patients with a skewed TCR Vβ repertoire amounted to 80.0 and 18.2 %, whereas in the elderly percentages were 100 % and 72.7 %, respectively (Fig. 2d). A similar trend occurred in the healthy population, only reaching significance (*P* < 0.01) in the elderly group (Fig. 2d). Although CMV latency significantly added to the skewing of the TCR Vβ repertoire in ESRD patients, the effect of ESRD on the TCR Vβ repertoire was present in both CMV-seropositive as well as CMV-seronegative elderly individuals. The percentage of elderly patients with a skewed TCR Vβ repertoire was significantly higher when compared to the CMV-status matched elderly HI (*P* < 0.05) (Fig. 2d).

### ESRD, aging, and CMV latency influenced the TCR Vβ repertoire diversity independently

To test the influence of several factors such as ESRD, aging, CMV latency, and gender (as the percentage of males was significantly higher in the patients compared to HI) on skewing of the TCR Vβ repertoire, a binary logistic regression model was used. Except for gender (*P* > 0.05), ESRD (*P* < 0.05, odds ratio (OR) = 4.8), age (*P* < 0.05, OR = 2.3) and CMV-IgG seropositivity (*P* < 0.05, OR = 6.7) were significantly associated with skewing of TCR Vβ repertoire. A multiple binary logistic regression model was used to further analyze whether the effects on TCR Vβ repertoire skewing were independent. ESRD (*P* < 0.05, OR =10.2), age (*P* < 0.05, OR =3.1) and CMV-IgG seropositivity (*P* < 0.05, OR =13.8) remained significant, indicating these factors to independently contribute to skewing of the TCR Vβ repertoire. CMV-IgG seropositivity contributed mostly to the skewing of the TCR Vβ repertoire followed by ESRD and age (Table 2). In the ESRD patients, neither renal replacement therapy (RRT) (*P* > 0.05) nor underlying kidney disease (*P* > 0.05) were associated with skewing of the TCR Vβ repertoire (Table 2).

**Table 2:** The variables related to a skewed TCR Vβ repertoire

	Non-skewed TCR Vβ	Skewed TCR Vβ	Odds ratio	*P* value
Age (years, median)	41	65	3.1^a^	< 0.05
% of CMV seropositivity	30.7	74.5	13.8^b^	< 0.05
% of ESRD patients	28.6	70.0	10.2^c^	< 0.05
% of patients on renal replacement therapy	35.8	48.4		NS
Underlying kidney disease (number)				
Nephrosclerosis/atherosclerosis/hypertensive nephropathy	1	7		NS
Primary glomerulopathy	0	3		NS
Diabetic nephropathy	2	7		NS
Reflux nephropathy	4	4		NS
Polycystic kidney disease	2	7		NS
Other	5	2		
Unknown	0	1		

*Abbreviations*: *TCR* T cell receptor, *NS* not significant

^a^means the odds of a skewed TCR Vβ repertoire in elderly population is 3.1 times higher than in the young population

^b^means the odds of a skewed TCR Vβ repertoire in CMV- IgG seropositive population is 13.8 times higher than in the CMV- IgG seronegative population

^C^means the odds of a skewed TCR Vβ repertoire in patients is 10.2 times higher than in the healthy individuals

### Skewed TCR Vβ repertoires are presented predominantly within the CD8^+^ memory T cell subset and is associated with more differentiated T cells

In order to determine whether skewing of TCR Vβ was associated with specific T cell subsets, CD4^+^/CD8^+^ naive and CD4^+^/CD8^+^ memory T cells from ten elderly healthy individuals (of which 50 % were CMV seropositive) as well as 10 elderly ESRD patients (also containing 50 % CMV-seropositive individuals) were FACS-sorted. Skewing of the TCR Vβ repertoire significantly occurred in CD8^+^, but not CD4^+^, memory T cells in elderly ESRD patients only (Table 3). Within the CD8 memory T cells, a skewed TCR Vβ repertoire was observed in 5 CMV-seropositive and 4 CMV-seronegative elderly ESRD patients.

**Table 3:** Frequency of elderly individuals with skewed and non-skewed TCR Vβ repertoire in the sorted T cells subsets

T cell subsets	HI			ESRD patients		
	Skewed	Non-skewed	*P* value	Skewed	Non-skewed	*P* value
CD4 naive	0^a^	10	NS	0	10	NS
CD4 Memory	2	8		3	7	
CD8 Naive	3	7	NS	3	7	< 0.05
CD8 Memory	7	3		9	1	

*Abbreviations*: *TCR* T cell receptor repertoire, *ESRD* end stage renal disease, *HI* healthy individuals, *NS* not significant

^a^Data are shown as the numbers of individuals

**Fig. 3:**
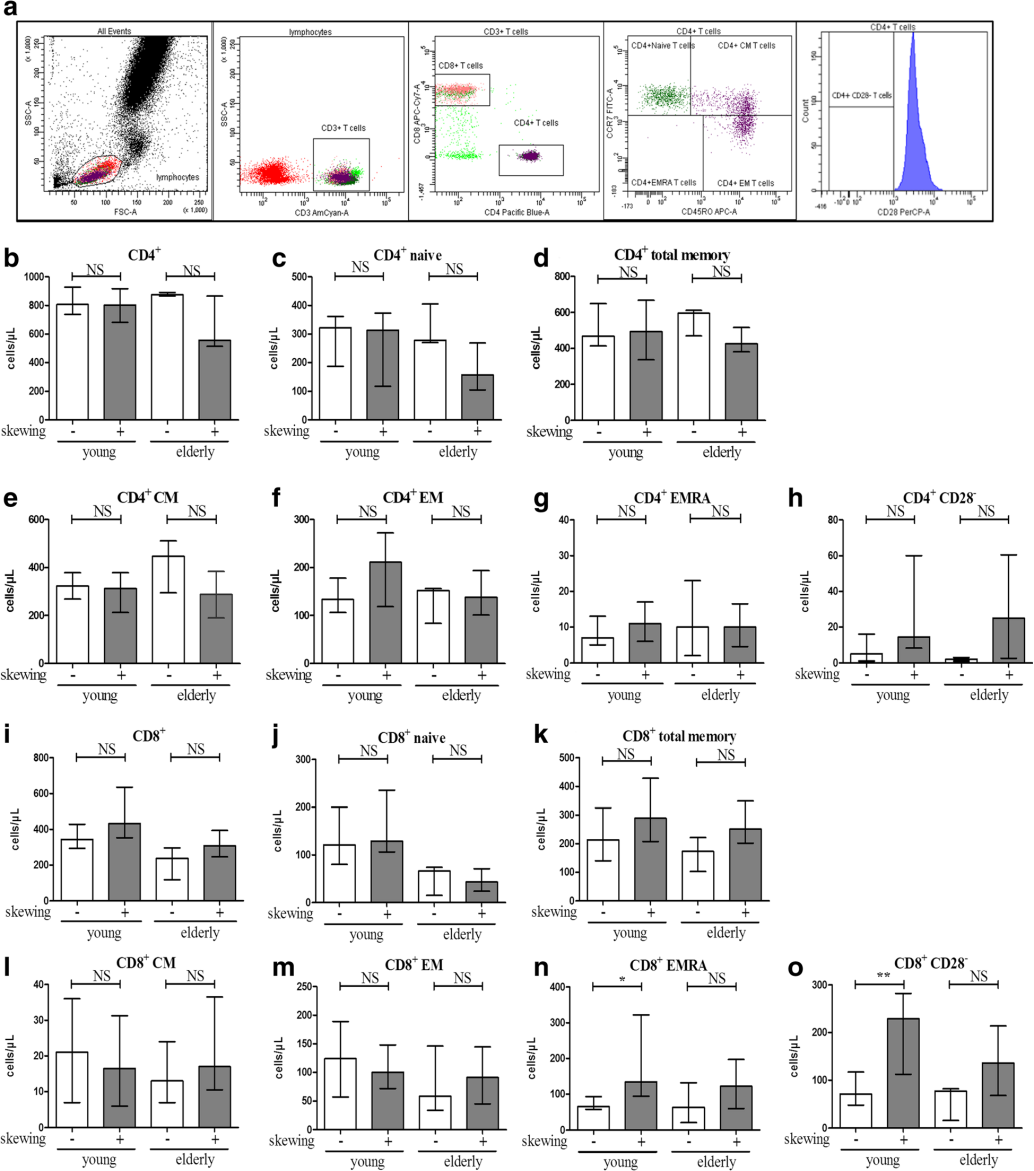
Absolute number of circulating CD4^+^ and CD8^+^ T cell subsets in young (*n* = 10) and elderly end stage renal disease (ESRD) patients (*n* = 21) with a skewed TCR Vβ repertoire compared to age-matched ESRD patients without a skewed TCR Vβ repertoire (young *n* = 11; elderly *n* = 3). A typical example of the gating strategy for dissection of the different T cell subsets by flow cytometry following a whole blood staining protocol is given in **a**. Briefly, lymphocytes were identified based on the forward/sideward characteristics followed by the selection of CD3^+^ T cells. These CD3^+^ T cells were then dissected into CD4^+^ and CD8^+^ T cells. CCR7 and CD45RO were used to identify the CD4^+^ T cells subsets. Furthermore, CD28^−^ T cells were examined within the total CD4^+^ population. A similar gating strategy was employed for determination of absolute numbers of CD8^+^ T cell subsets and the differentiation status. The number of (**b**) CD4^+^, (**c**) CD4^+^ naive, (**d**) CD4^+^ total memory, (**e**) CD4^+^ central memory (CM), (**f**) CD4^+^ effector memory (EM), (**g**) CD4^+^ highly differentiated effector T cells (EMRA), (**h**) CD4^+^CD28^−^ T cells, as well as (**i**) CD8^+^, (**j**) CD8^+^ naive, (**k**) CD8^+^ total memory, (**l**) CD8^+^ central memory (CM), (**m**) CD8^+^ effector memory (EM), (**n**) CD8^+^ highly differentiated effector T cells (EMRA) and (**o**) CD8^+^CD28^−^ T cells are shown. Data are given as median with interquartile range. The open bars represent ESRD patients with a non-skewed TCR Vβ repertoire and closed bars correspond to ESRD patients with a skewed TCR Vβ repertoire. P value: * < 0.05; ** < 0.01; *** < 0.001; NS: not significant

The ratio of CD4^+^/CD8^+^ T cells was significantly lower in young and elderly ESRD patients with a skewed TCR Vβ repertoire compared to age-matched ESRD patients without skewed TCR Vβ repertoire, respectively (young: 1.73 versus 2.38 *P* < 0.05; elderly: 2.30 versus 4.71, *P* < 0.05). Next, we determined the association between having a skewed TCR Vβ repertoire and T cell subsets as well as the differentiation status of T cells. In Fig. 3a, a typical flow cytometric example of the gating strategy for dissecting the different CD4^+^ T-cell subsets including naive, central memory (CM), effector memory (EM), highly differentiated effector memory (EMRA) as well as for determining the differentiation status of the T-cell compartment by analysis of CD28^−^ T cells is given. A similar approach was also employed for CD8^+^ T cells. Young ESRD patients with a skewed TCR Vβ repertoire had increased numbers of more differentiated memory T cells, i.e. EMRA (Fig. 3n) as well as CD28^−^CD8^+^ T cells (Fig. 3o). The number of individuals without skewing in the elderly ESRD patients was limited (only 3 out of 24), which might explain why the comparison between non-skewed and skewed ESRD patients did not reach significance for CD8 EMRA or CD8 CD28- T cells but similar trends were observed (Fig. 3n & o). Approximately 80 % of the young and 62 % of the elderly ESRD patients, that had a skewed TCR Vβ repertoire, were CMV-seropositive, confirming the previously described effects of CMV-latency on the T cell compartment. Moreover, the skewed TCR Vβ repertoire was correlated with more differentiated CD8^+^ T cells in the healthy population as well. In the elderly healthy individuals, those with a skewed TCR Vβ repertoire had more CD8^+^ T cells, more CD8^+^ memory, CD8^+^ EMRA and CD8^+^CD28^−^ than the individuals without skewed TCR Vβ repertoire (Additional file 1: Figure S1 H, J, M, and N). Within young healthy individuals, the numbers of CD8^+^ memory (MEM), CD8^+^ effector memory (EM) and CD8^+^CD28^−^ T cells were significantly higher in the individuals with skewed TCR repertoire than the individuals with a polyclonal TCR Vβ repertoire (Additional file 1: Figure S1 J, L, N). The proportions of CMV-IgG seropositive individuals within the HI with a skewed TCR repertoire amounted to 75 % and 100 % in young and elderly HI, respectively.

### Number of CD31^+^ naive T cells and relative telomere length (RTL) are not correlated to a skewed TCR Vβ repertoire

**Fig. 4:**
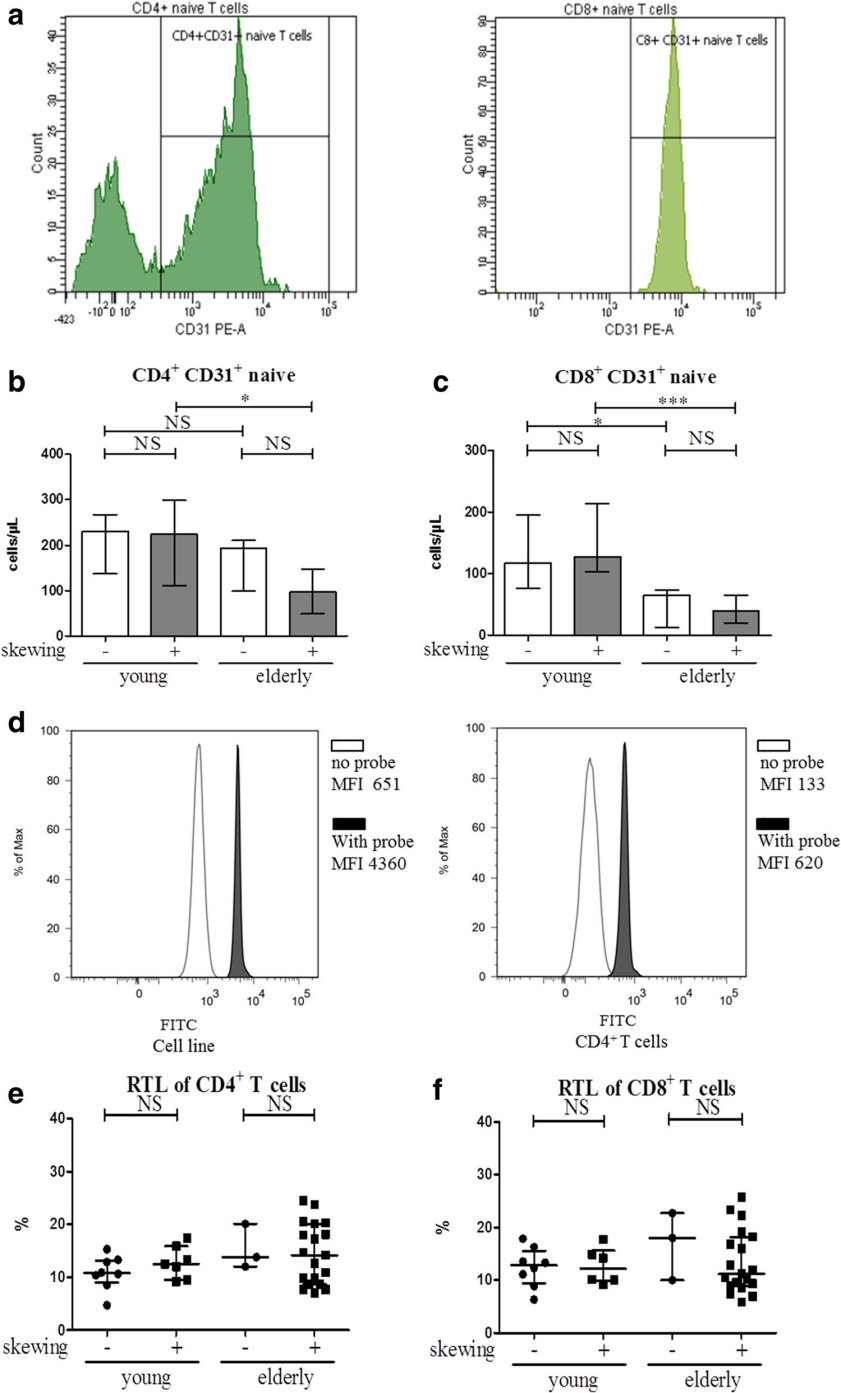
Absolute number of CD31-expressing naive CD4^+^ and CD8^+^ T cells and relative telomere length (RTL) of CD4^+^ and CD8^+^ T cells in end stage renal disease (ESRD) patients with skewing in TCR Vβ repertoire compared with age-matched ESRD patients without skewing in TCR Vβ repertoire. A typical flow cytometric example of the analysis of CD31-expression within naive CD4^+^ (*left histogram*) and CD8^+^ (*right histogram*) T cells (identified as depicted within Fig. 3a) is given in **a**. The number of CD31^+^ (**b**) CD4^+^ and (**c**) CD8^+^ naive T cells for young and old ESRD patients with a non- (*open bars*; young *n* = 11, elderly *n* = 3) and skewed (*closed bars*; young *n* = 10, elderly *n* = 21) TCR Vβ repertoire are given. In **d**, a typical flow cytometric example is depicted of the analysis to allow for the determination of relative telomere length (RTL). Histograms showing the median fluorescence intensity (MFI) of the FITC-channel of samples incubated without (*unfilled peak*) and with the FITC-labelled PNA-probe (*filled peak*) are shown for the CCRF-CEM 1301 subcell-line (*left panel*) and CD4^+^ (*right panel*), respectively. The RTL of (**e**) CD4^+^ and (**f**) CD8^+^ T cells are shown. Triangles represent the RTL of ESRD patients with a non-skewed TCR Vβ repertoire (young = 8, elderly *n* = 3) and squares correspond to ESRD patients with a skewed TCR Vβ repertoire (young *n* = 6, elderly *n* = 19). Data are given as median with interquartile range. * < 0.05; ** < 0.01; *** < 0.001; NS: not significant

Thymic output, assessed by number of CD31-expressing naive CD4^+^ and CD8^+^ T cells (Fig. 4a), was not different for ESRD patients or healthy individuals with and without a skewed TCR Vβ repertoire. Remarkably, only elderly patients and HI with a skewed TCR Vβ repertoire showed an age-related decline in CD31^+^ naive CD4^+^ T cells (Fig. 4b and Additional file 1: Figure S2A). An age- related decline in CD31^+^ naive CD8^+^ T cells was observed in both patients and HI (Fig. 4c and Additional file 1: Figure S2B). Another hallmark of T cell ageing is attrition of telomeres and a typical example of the flow cytometric analysis of telomere length is shown in Fig. 4d. Assessing the association of this parameter to skewing of the TCR Vβ repertoire only revealed a trend to shorter telomeres in CD8^+^ (Fig. 4f) T cells of elderly ESRD patients with a skewed TCR Vβ repertoire compared to those without. The RTL of CD4^+^ /CD8^+^ T cells were not significantly different between the healthy individuals with and without a skewed TCR repertoire either (Additional file 1: Figure S2 C&D).

## Discussion

The main observation of this study is that ESRD independently skews the TCR Vβ repertoire. In combination with an older age and CMV seropositivity, this leads to a skewed TCR repertoire in almost all elderly ESRD patients. This finding extends the knowledge that loss of renal function is associated with significant changes in several T-cell (aging) parameters such as thymic output, T-cell differentiation status and proliferative history as analyzed by relative telomere length analysis [6, 7]. In contrast with these studies that demonstrate effects of ESRD on various T-cell parameters over a broad age range, skewing of the TCR Vβ repertoire by ESRD is limited to the elderly, which indicates that young ESRD patients are still able to maintain a relatively diverse TCR Vβ repertoire.

In our study, we tested all TCR Vβ gene families, in a multiplex DNA-based approach and considered different variables possibly influencing TCR Vβ repertoire diversity, like age [13, 22], CMV-serostatus [17], gender [23], ESRD, RRT and underlying kidney disease [24, 25]. Although CMV was the strongest factor amongst the examined variables, affecting both the young and elderly ESRD patients as well as elderly HI, ESRD seems to introduce skewing in both the CMV-seronegative as well as CMV-seropositive elderly group. We only observed an age-related effect on TCR Vβ skewing in our ESRD patient population, but aging alone does not seem to significantly compromise TCR Vβ skewing. It is also reported that the TCR-Vβ repertoire of CD8^+^ T cells was already skewed in young CMV-seropositive HI and did not modulate further with age [26]. Also, Sunder-Plassmann et al. [27] showed ESRD patients receiving hemodialysis treatment to have a skewed TCR Vβ repertoire, with an impressive increase of TCR Vβ6.7 and a massive deletion of the TCR Vβ8 in peripheral blood lymphocytes compared to healthy controls. In our study, we were unable to find a significant association between RRT and skewing of the TCR Vβ repertoire. This is in accordance with our previous findings, which did show a significant effect of ESRD on ageing of the peripheral T cells but hardly any differences between patients with or without RRT. [8] Gender also did not affect TCR Vβ skewing and in agreement with the research by Sunder-Plassmann et al. [27], we did not find a significant contribution of underlying kidney disease with respect to skewing of the TCR Vβ repertoire. This might indicate that the chronic inflammatory milieu [28] in which the T cells circulate and the significant changes in the peripheral T cell compartment [6] as a consequence of the loss of renal function causes oligoclonality of the TCR repertoire.

A skewed TCR Vβ repertoire is associated with significant changes in composition of peripheral T cells and related to a highly differentiated pattern. A skewed TCR Vβ repertoire in HI was correlated to an increased number of highly differentiated memory CD8^+^ T cells. This might be due to the fact that most of the healthy individuals with a skewed TCR Vβ repertoire were CMV-seropositive. CMV latency introduces significant changes in the peripheral T cell compartment, in particular affecting CD8^+^ T cells [29] and is known to introduce skewing of the TCR Vβ repertoire as a result of expansion of CMV-specific T cell clones in the HI [17]. In refractory cytopenia patients, TCR Vβ skewing correlates with an expansion of effector CD8+ T cells [30]. A similar correlation between a skewed TCR Vβ repertoire and an increased number of highly differentiated memory CD8^+^ T cells was also found in young ESRD patients and tended to be significant in the elderly patients. Also within ESRD patients, a large proportion was CMV-IgG seropositive, highlighting the possibility that skewing of TCR Vβ repertoire may be likewise related to clonal expansions of antigen-specific T cells directed to CMV or expansion of other TCR-specificities, e.g. TCR Vβ 6.7 as reported before [27].

CD31-expressing naive T cells have undergone a lower number of cell divisions [10] and have a broader TCR Vβ repertoire than those lacking CD31 [11]. Some clones could be lost during naïve T cell homeostatic proliferation [31], as a massive deletion of the TCR Vβ8 was observed in peripheral blood lymphocytes of hemodialysis patients [27]. However, the numbers of CD31- expressing naïve T cells were not significantly different between the patients with skewed and non-skewed TCR Vβ repertoire. In addition, the skewed TCR Vβ repertoire was predominantly observed in the sorted CD8^+^ memory compartment and not in naïve T cells. This implies that some memory T-cell clones proliferate more efficiently in response to the ESRD associated pro-inflammatory environment or antigenic stimulation such as CMV. Clonal expanded CD8^+^ T cells are also observed in the peripheral blood of multiple sclerosis patients. Based on the current findings, we plan to conduct a more in depth analysis of the TCR Vβ repertoire including both the diversity (the number of specific clones) and the relative abundance of particular clones (the number of reads per clone). The clinical implications of having a skewed or less diverse TCR Vβ repertoire prior to kidney transplantation are not yet explored. However, analysis of TCR Vβ repertoire diversity following transplantation has been the subject of several studies [32, 33]. Miqueu et al. [34] reported a skewed repertoire to be related to an increased risk of humoral chronic rejection after kidney transplantation. It might be of interest to investigate the consequences of a skewed TCR Vβ repertoire before kidney transplantation with respect to clinical outcomes following kidney transplantation like rejection episodes or to examine whether this TCR Vβ repertoire would change after kidney transplantation.

## Conclusions

ESRD significantly and independently skews the TCR Vβ repertoire in the elderly individuals and this skewed TCR Vβ pattern is associated with more differentiated T cells subsets. Assessing the TCR Vβ repertoire diversity in more detail will increase our knowledge with respect to the defective T-cell mediated immunity observed in ESRD patients and will allow to evaluate its clinical relevance with respect to transplantation.

## Methods

### Study population

Forty-five stable adult ESRD patients, defined as a glomerular filtration rate of ≤ 15 ml/min with or without hemodialysis, on the waiting list for the first kidney transplantation and 51 healthy kidney donors were included (Table 1) from 1^st^ November 2010 to 1^st^ October 2013 in nephrology department of Erasmus medical center. Patients with any clinical or laboratory evidence of acute bacterial or viral infection, malignancy, previous kidney transplantations, immunosuppressive drugs treatment within 28 days prior to transplantation (except glucocorticoids) were excluded. Age, CMV-IgG serostatus and total T-cell number in the peripheral blood were matched between these two groups. Lithium-heparinized blood was drawn prior to transplantation of ESRD patients and healthy kidney donors. All individuals included gave informed consent and the local medical ethical committee approved the study (METC number: 2012–022). It was conducted according to the principles of Declaration of Helsinki and in compliance with International Conference on Harmonization/Good Clinical Practice regulations.

### Circulating T cell numbers and their differentiation status

Freshly drawn peripheral blood samples from 45 ESRD patients and 51 healthy individuals (HI) were stained and acquired on a FACSCanto II flow cytometer (BD Biosciences, Erembodegem, Belgium) as described previously [7, 35] to determine both frequencies and absolute numbers of the different T cell subsets as well as their differentiation status. Data were analyzed using FACS Diva software version 6.1.2 (BD Biosciences). Detailed methods can be found in the Additional file 1: Methods section.

### PBMCs isolation, DNA isolation and TCR Vβ repertoire analysis

PBMCs from 45 ESRD patients and 51 healthy individuals (HI) were isolated from peripheral blood as described previously [3]. One million PBMCs were snap-frozen for DNA isolation and the remaining were frozen at 10 million per vial until further use. DNA was isolated according to manufacturer’s instructions (QIAamp DNA Mini QIAcube Kit). The TCR Vβ gene repertoire was measured by the multiplex TCR Vβ-Jβ gene PCR as developed and approved by the European consortium of 45 laboratories (BIOMED-2 Concerted Action BMH4-CT98-3936) [21]. The term skewed TCR Vβ repertoire was used when the TCR Vβ showed an oligoclonal pattern with one or more clonal peak(s) on the Genescan profile and the term non-skewed Vβ repertoire was used when the spectratype of TCR Vβ repertoire on the Genescan profile showed a Gaussian distribution [21].

### Sorting of T cell subsets

Cryopreserved PBMCs were thawed, washed and resuspended in PBS. The PBMCs from ten elderly patients (of which 50 % were CMV-seropositive) and 10 elderly healthy individuals (of which 50 % were CMV-seropositive) were stained and subsequently sorted into T cells subsets i.e. CD4^+^ naive/memory and CD8^+^ naive/ memory using flow cytometry based cell-sorting (BD FACSAria™ II SORP, BD). A typical example of the gating strategy to dissect the different T cell subsets prior to sorting as well as the analysis of the purity of the sorted samples was given in Additional file 1: Figure S3. The purity of all T cell subsets was over 95 %. The details for flow cytometry-based cell-sorting of T cell subsets are given in the Additional file 1: Methods section.

### Telomere length assay

Flow fluorescent in situ hybridization was performed to determine the relative telomere length (RTL) of T cells as described previously [7, 35]. Detailed information is given in the Additional file 1: Methods section.

### Statistical analyses

Statistical analyses were performed using SPSS 20 (IBM, Chicago, IL, USA) and GraphPad Prism 6 (GraphPad Software, Inc., La Jolla, CA, USA). Categorical variables were compared using the chi-square test or Fisher’s exact test. Continuous variables were compared using Mann–Whitney U-test. Binary logistic regression was used to estimate the probability that skewing of TCR repertoire was present given the values of explanatory variables. All reported P-values are two-sided and were considered statistically significant when *P* < 0.05.

## Additional file

Additional file 1: **Figure S1.** Absolute number of circulating CD4^+^ and CD8^+^ T cells in young (*n* = 8) and elderly healthy individuals (HI, *n* = 8) with skewing in TCR Vβ repertoire compared to age-matched HI without skewing in TCR Vβ repertoire (young *n* = 18; elderly *n* = 17). **Figure S2**. Absolute number of CD31-expressing naive and relative telomere length (RTL) of CD4^+^ and CD8^+^ T cells of healthy individuals (HI) with skewing in TCR Vβ repertoire compared to age-match HI without skewing in TCR Vβ repertoire. **Figure S3**. A typical example of the gating strategy to dissect the different T cell subsets prior to sorting (A) and the analysis of the purity of CD4^+^ naive/memory sorted samples (B). Supplementary materials and methods. (PDF 83890 kb)

## Abbreviations

CM: Central Memory
CMV: Cytomegalovirus
EM: Effector Memory
EMRA: Effector Memory CD45RA^+^
ESRD: End-Stage Renal Disease
FITC: Fluorescein isothiocyanate
HI: Healthy individuals
PB: Pacific blue
PBMCs: Peripheral Blood Mononuclear Cells
PE: Phycoerythrin
PerCpCy5.5: Peridinin chlorophyll-Cy5.5
RTL: Relative Telomere Length
TCR: T cell receptor
