# Supplementary material for: End stage renal disease patients have a skewed T cell receptor Vβ repertoire
Source: Immun Ageing. 2015 Dec 14;12:28. doi: 10.1186/s12979-015-0055-7 (PMC4681023; doi:10.1186/s12979-015-0055-7)
Supplement: Supplementary file 1 — Figure S1. Absolute number of circulating CD4+ and CD8+ T cells in young (n = 8) and elderly healthy individuals (HI, n = 8) with skewing in TCR Vβ repertoire compared to age-matched HI without skewing in TCR Vβ repertoire (young n = 18; elderly n = 17). Figure S2. Absolute number of CD31-expressing naive and relative telomere length (RTL) of CD4+ and CD8+ T cells of healthy individuals (HI) with skewing in TCR Vβ repertoire compared to age-match HI without skewing in TCR Vβ repertoire. Figure S3. A typical example of the gating strategy to dissect the different T cell subsets prior to sorting (A) and the analysis of the purity of CD4+ naive/memory sorted samples (B). Supplementary materials and methods. (PDF 83890 kb) [file 12979_2015_55_MOESM1_ESM.pdf]

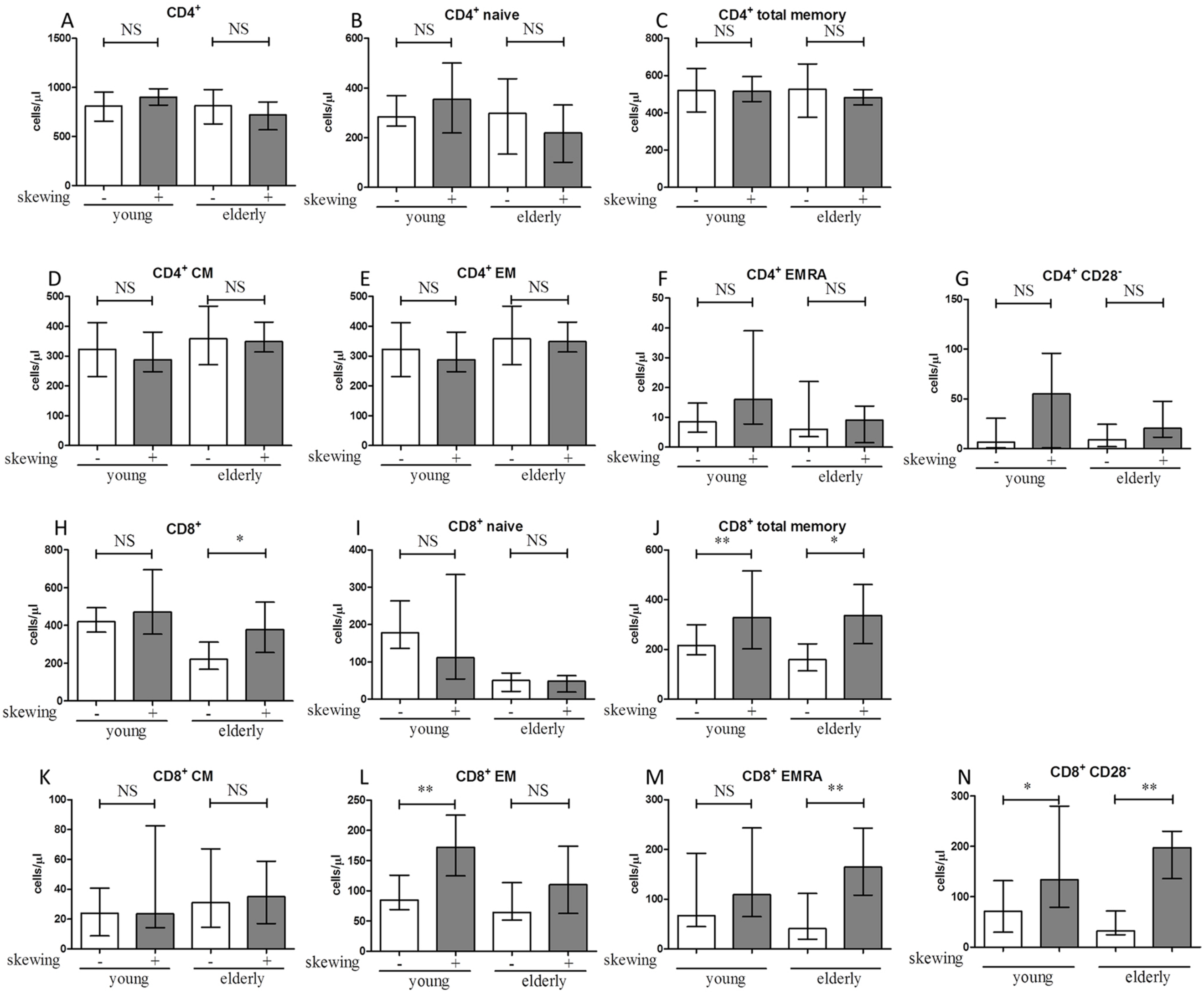

Supplementary Figure 1. Absolute number of circulating CD4$^+$ and CD8$^+$ T cells in young (n=8) and elderly healthy individuals (HI, n=8) with skewing in TCR Vβ repertoire compared to age-matched HI without a skewing in TCR Vβ repertoire (young n=18; elderly n=17). The number of (**A**) CD4$^+$, (**B**) CD4$^+$ naive, (**C**) CD4$^+$ total memory, (**D**) CD4$^+$ central memory (CM), (**E**) CD4$^+$ effector memory (EM), (**F**) CD4$^+$ highly differentiated effector T cells (EMRA), (**G**) CD4$^+$ CD28$^-$ T cells, as well as (**H**) CD8$^+$, (**I**) CD8$^+$ naive, (**J**) CD8$^+$ total memory, (**K**) CD8$^+$ central memory (CM), (**L**) CD8$^+$ effector memory (EM), (**M**) CD8$^+$ highly differentiated effector T cells (EMRA) and (**N**) CD8$^+$ CD28$^-$ T cells are shown. Data are given in median with interquartile range. Open bars represent HI with a non-skewed TCR Vβ repertoire and closed bars correspond to HI with a skewed TCR Vβ repertoire. *<0.05, **<0.01, ***<0.001; NS: not significant.

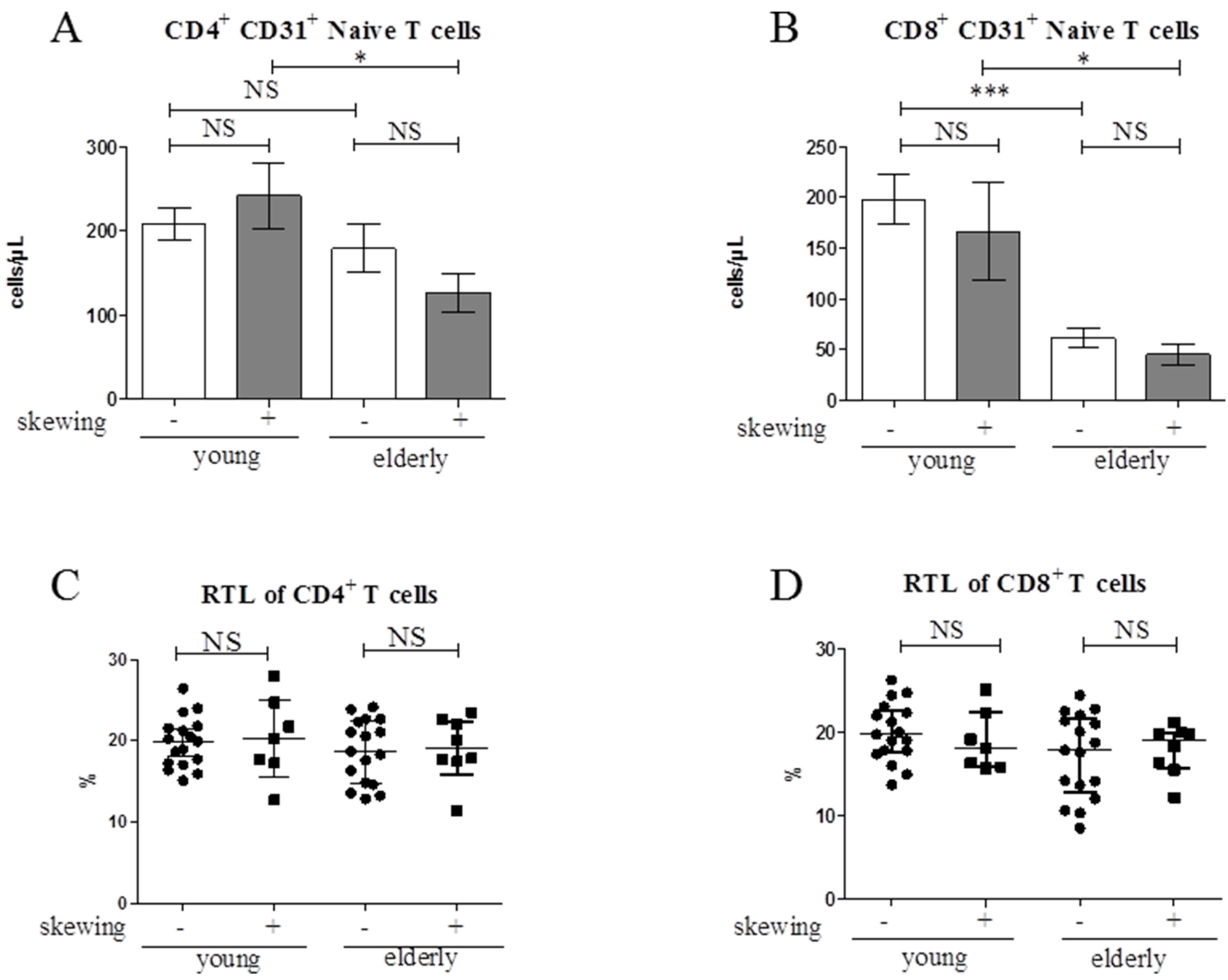

Supplementary Figure 2. **Absolute number of CD31-expressing naïve and relative telomere length (RTL) of $CD4^+$ and $CD8^+$ T cells of healthy individuals (HI) with skewing in TCR Vβ repertoire compared to age-match HI without skewing in TCR Vβ repertoire.** Number of (A) $CD31^+$ $CD4^+$ naïve and (B) $CD31^+$ $CD8^+$ naïve T cells, in addition with RTL of (C) $CD4^+$ and (D) $CD8^+$ T cells are shown. Data are given in median with interquartile range. Open bars represent absolute number of CD31- expressing naïve T cells of HI with a non-skewed TCR Vβ repertoire (young n=18; elderly n=17) and closed bars correspond patients with a skewed TCR Vβ repertoire (young n=8, elderly n=8). Triangles represent the RTL of patients with a non-skewed TCR Vβ repertoire (young=17, n=17) and squares correspond patients with a skewed TCR Vβ repertoire (young n=7, elderly n=8). *<0.05; **<0.01; ***<0.001; NS: not significant

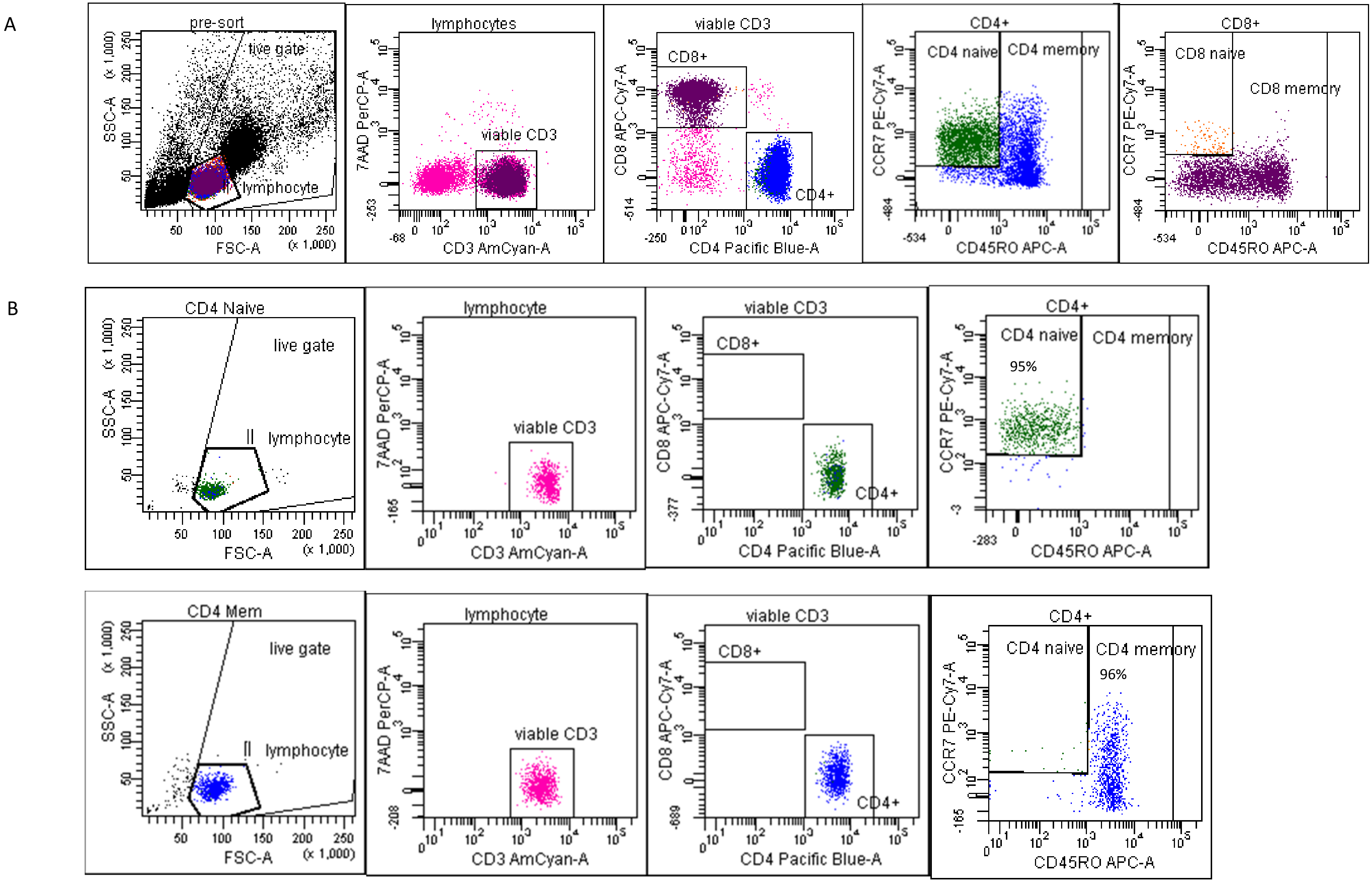

Supplementary Figure 3. A typical example of the gating strategy to dissect the different T cell subsets prior to sorting (A) and the analysis of the purity of CD4$^+$ naive/memory sorted samples (B). Purity is determined by assessing the frequency of a particular T cell subset within the live gate of that sorted samples, i.e. the frequency of CD4$^+$ naive T cells within total live cells in the naive-sorted sample.

A

B

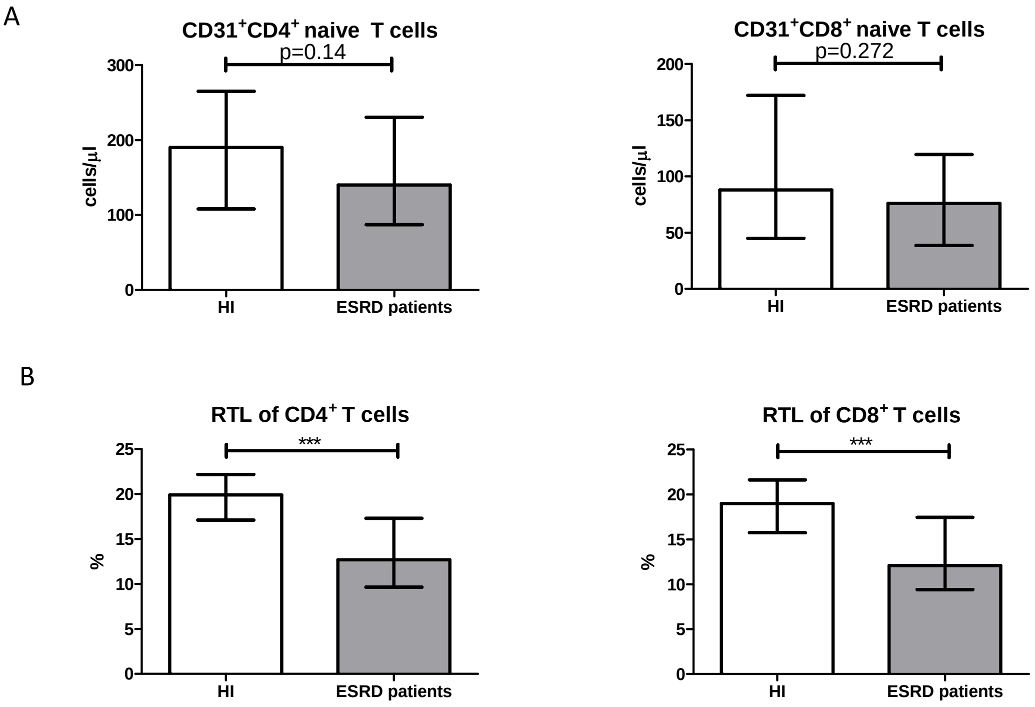

Supplementary Figure 4. Numbers of CD31-expressing T cells between the ESRD patients (N=45) and healthy individuals (N=51) (A) and relative telomere length (RTL) between ESRD patients (N=37) and HI (n=49) (B). Open bars represent HI and closed bars correspond to HI. Data are given as median with interquartile range. P values are given and *** means the P value is lower than 0.001.

Supplementary materials and methods

Circulating T cell numbers and their differentiation status

Absolute numbers of T cells were obtained using BD TruCount ™ tubes. Briefly, 20 µl of the 6-color TBNK reagent (BD Multitest™, BD Bioscience) was used in combination with a Trucount™ tube (BD Biosciences) and 50 µl of EDTA blood. This tube contains a fixed number of beads (i.e. bead count; lot-specific) which enables calculation of absolute numbers of cells per µl of blood. The 6-color TBNK reagent contains fluorescein isothiocyanate (FITC)-labeled anti-CD3, phycoerythrin (PE) labeled anti-CD16/CD56, peridinin chlorophyll protein **(**PerCP) - cyanine dye (CY) ™5.5-labeled anti-CD45, PE-Cy™7- labeled anti-CD4, allophycocyanin (APC) - labeled anti-CD19, APC-Cy7 labeled anti-CD8.

Analysis of the T cell differentiation status was performed using flow-cytometry following the whole blood staining protocol. Briefly, whole blood was stained with AmCyan-labeled anti-CD3 (BD) in combination with pacific blue (PB)-labeled anti-CD4 (BD) and APC-Cy7-labeled anti-CD8 (BD). T cells were defined as $CD4^+$ or $CD8^+$ and further defined into four different subsets based on the expression of CCR7 and CD45RO using FITC- labeled anti-CCR7 (R&D systems, Uithoorn, The Netherlands) and APC-labeled anti-CD45RO (BD). Naive T cells were identified as $CCR7^+$ and $CD45RO^-$, central memory (CM) cells as $CCR7^+$ and $CD45RO^+$, effector memory (EM) cells as $CCR7^-$ and $CD45RO^+$ and the highly differentiated effector memory (EMRA) cells as $CCR7^-$ and $CD45RO^-$. T-cell differentiation is associated with loss of CD28 expression on the cell surface. Numbers of $CD28^-$ (or CD28null) T cells within the T-cell subsets were determined by staining with PerCP-Cy5.5-labeled anti-CD28 (BD). Thymic output was analyzed by determining numbers of CD31-expressing cells within the naive T-cell pool

upon staining with PE-labeled anti-CD31 (Biolegend, Europe BV, Uithoorn, the Netherlands). Samples were measured on the FACSCanto II (BD) acquiring at least $5x10^4$ T lymphocytes.

Sorting of T cell subsets

PBMCs were stained with AmCyan-labeled CD3 (BD Pharmingen, Erembodegem, Belgium), Pacific Blue-labeled CD4 (BD), APC-Cy7- labeled CD8 (BD); APC-labeled CD45RO (BD), PE-CY7-labeled CCR7 (R&D systems, Uithoorn, The Netherlands) and a live-dead marker ViaProbe (7-Aminoactinomycin D, BD) was included. After staining, the cells were washed and resuspended at $20\text{-}25x10^6$/mL and sorted into CD4+ and CD8+ Naïve/memory T cells (BD FACSAria™ II SORP, BD). Naive T cells were identified as CCR7+ and CD45RO- and the remaining CD4+ or CD8+ T cells were collected as memory T cells.

Telomere length assay

Briefly, PBMCs were thawed and stained with either anti-CD4-biotin (Beckman-Coulter, BV, Woerden, the Netherlands) or anti-CD8-biotin (Biolegend) followed by staining with streptavidin-Cy5 (Biolegend). After fixation and permeabilization, the telomere length of T cells was determined using the telomere PNA-kit/FITC (Dako BV, Heverlee, Belgium) according to manufacturer's instructions. The sub-cell line 1301 of CCRF-CEM, known for its long telomeres was treated in a similar way and used as an internal control. The relative telomere length was calculated using the following formula, comparing the median fluorescence intensity (MFI) of the FITC signal of the samples to that of the sub-cell line.

$$\text{RTL}=\frac{(\text{MFI of sample cells with probe} - \text{MFI of sample cells without probe})\text{x DNA index of control cells }(=2)}{(\text{MFI of cell line with probe} - \text{ MFI of cell line without probe})\text{ x DNA index of sample cells}(=1)}*100\%$$
